# Highly Efficient Elimination of Carbon Monoxide with Binary Copper-Manganese Oxide Contained Ordered Nanoporous Silicas

**DOI:** 10.1186/s11671-015-1197-4

**Published:** 2016-01-07

**Authors:** Jiho Lee, Hwayoun Kim, Hyesun Lee, Seojun Jang, Jeong Ho Chang

**Affiliations:** Korea Institute of Ceramic Engineering and Technology, Gyengnam, 660-031 South Korea

**Keywords:** Nanoporous, Catalysts, Carbon monoxide, Elimination, Copper-manganese oxide

## Abstract

Ordered nanoporous silicas containing various binary copper-manganese oxides were prepared as catalytic systems for effective carbon monoxide elimination. The carbon monoxide elimination efficiency was demonstrated as a function of the [Mn]/[Cu] ratio and reaction time. The prepared catalysts were characterized by Brunauer-Emmett-Teller (BET) method, small- and wide-angle X-ray diffraction (XRD), and high-resolution transmission electron microscopy (HR-TEM) for structural analysis. Moreover, quantitative analysis of the binary metal oxides within the nanoporous silica was achieved by inductively coupled plasma (ICP). The binary metal oxide-loaded nanoporous silica showed high room temperature catalytic efficiency with over 98 % elimination of carbon monoxide at higher concentration ratio of [Mn]/[Cu].

**Electronic supplementary material:**

The online version of this article (doi:10.1186/s11671-015-1197-4) contains supplementary material, which is available to authorized users.

## Background

Methods to effectively eliminate carbon monoxide have attracted much attention [1–5]. Recently, the related materials have been reported by different groups. It would be better to cite some examples [6–8]. Even though these supported noble metal-based catalysts have shown high activities for carbon monoxide elimination, their further application has been limited due to difficulties in reuse, sintering at high temperature, and high cost [9–12]. For this reason, the development of transition metal oxide catalysts as alternatives has gained much interest.

Transition metal oxides like CuO_x_, MnO_x_, and FeO_x_ have so far been used for the elimination of carbon monoxide in bimetallic forms [13–15]. Recently, the related materials have been reported by different groups. It would be better to cite some examples [16, 17]. The binary Cu-Mn oxides have flexible metal valences (Cu^1+^/^2+^ and Mn^3+^/^4+^) which give rise to their specific properties and notable catalytic activities in carbon monoxide elimination [18, 19]. In particular, the incomplete Mn_1.5_Cu_1.5_O_4_ spinel structure of the binary Cu-Mn oxide catalyst was more active in the removal of carbon monoxide at room temperature than that of the same spinel structure with CuO [19–21]. In addition, its level of activity in removing carbon monoxide was reduced if the catalyst was calcined at a temperature above 500 °C, at which the crystallization of the spinel occurs.

Porous materials are typically used for separation, biological immobilization, catalysts, and supports, because of their high surface area and unique physical and chemical properties [22–27]. Porous materials such as zeolites have also been employed as supports for metal oxide nanoparticles for the removal of carbon monoxide [28, 29]. However, such materials limit the incorporation of nanoparticles into the micropores, as well as the diffusion of the reactant, due to irregular micropores. For these reasons, ordered porous structures with high surface area and mesopore size, such as MCM-41 and SBA-15, have been widely used as catalyst supports [26, 27]. Consequently, various metal oxide-loaded mesoporous silica catalysts, such as Fe/SBA-15, CuO/SBA-15, and CuO-CeO_2_/SBA-15, have been studied and observed to provide improved performance in carbon monoxide elimination [23, 30, 33]. However, as yet, there has been little study of binary CuMnO_x_-loaded mesoporous silica for the elimination of gaseous phase carbon monoxide.

Herein, we report on binary CuMnO_x_-loaded mesoporous silica catalysts, prepared using a co-precipitation method, and their catalytic performance for gaseous carbon monoxide elimination, achieved at ambient temperature with various types of [Mn]/[Cu] ratios. This co-precipitation method allowed for room temperature synthesis of amorphous binary CuMnO_x_ with high catalytic activity for CO elimination at room temperature. The CO elimination results demonstrate that CuMnO_x_@MS-4 (with a [Mn]/[Cu] volume ratio of 4/1) can efficiently achieve >98 % elimination of CO gas within 420 min at room temperature.

## Methods

### Materials

Poly(ethylene oxide)-b-poly(propylene oxide)-b-poly(ethylene) triblock copolymer (Pluronic P123, PEO_20_PPO_70_PEO_20_), hydrochloric acid, tetraethyl orthosilicate (TEOS), manganese nitrate hexahydrate, and copper nitrate trihydrate were used from Sigma-Aldrich. All chemicals were used as received without any further purification.

### Synthesis of Ordered Mesoporous Silica (MS)

The ordered mesoporous silica support was synthesized following our previously reported method [23, 24]. Tri-block copolymer Pluronic P123 was dissolved in aqueous hydrochloric acid solution (1 < pH < 2) under vigorous stirring at 40 °C. A clear solution was obtained by incubating a complete dissolution of the surfactant. The tetraethyl orthosilicate (TEOS) was added into the solution at 40 °C as a silica source. The mixture was aged in a stainless steel bomb at 120 °C overnight. The precipitate was filtered, washed with excess water, air-dried at room temperature, and calcined at 550 °C.

### Synthesis of Binary Metal Oxide-Loaded Ordered Mesoporous Silica Catalysts (CuMnO_x_@MS)

The CuMnO_x_@MS catalysts were prepared by co-precipitation method at ambient temperature with an aqueous solution of Cu(NO_3_)_2_ and Mn(NO_3_)_2_. An aqueous solution of Cu(NO_3_)_2_°3H_2_O (0.25 M) and Mn(NO_3_)_2_°6H_2_O (0.25 M) was pre-mixed and impregnated into the mesoporous silica. The CuMnO_x_@MS catalysts were synthesized as a function of various molar ratios of [Mn]/[Cu]. The compositions were in the range of [Mn]/[Cu] 1/1 (CuMnO_x_@MS-1), 2/1 (CuMnO_x_@MS-2), and 4/1 (CuMnO_x_@MS-4), respectively. Subsequently, an aqueous solution of Na_2_CO_3_ (2 M) was added to maintain the pH at 8. The composite was aged for 2 h and heated to 80 °C. The composite was recovered by filtration and washed several times with hot deionized water, air-dried at room temperature, and calcined at 400 °C for 2 h.

### Characterization

Small-angle X-ray scattering (SAXS) patterns were obtained on a Rigaku DMAX-2500 diffractometer using Cu-K_*α*_ radiation (λ = 0.15418 nm) at 40 kV and 20 mA. The SAXS measurements were collected in the range 0.5°–4° of 2θ with a scanning speed of 2°min^−1^. Wide-angle X-ray diffraction (WAXD) patterns were recorded using a Rigaku DMAX-2500 Instrument with Cu-K_*α*_ radiation. The samples were scanned in the range 20°–80° of 2θ with a scanning speed of 2°min^−1^.

Nitrogen adsorption-desorption isotherms were obtained with a Micromeritics TriStar II system. The Brunauer-Emmett-Teller (BET) method was utilized to calculate the surface areas. The pore size distribution curves were obtained from the desorption branch calculated by the Barrett-Joyner-Halenda (BJH) method. The morphological and structural details of the material were also studied by field emission scanning electron microscopy (FE-SEM) and high-resolution transmission electron microscopy (HR-TEM). FE-SEM investigations were carried out with a JEOL JSM-6700 F instrument using 10 kV of accelerating voltage. Energy-dispersive X-ray spectroscopy (EDX) attached to the electron microscopy was used to qualitatively determine the elements present. HR-TEM was carried out on a JEOL JEM-4010 electron microscope operated at 400 kV. Cross-sectional slices of CuMnO_x_@MS, less than 60 nm in thickness, were prepared by using an ultramicrotome. To determine Cu, Mn, and Si ion contents in the various catalysts, the dried samples were weighed and digested with a mixed solution of phosphoric acid and ammonium metavanadate solution in sulfuric acid and hydrofluoric acid by heating. And then the Cu, Mn, and Si contents were analyzed, using inductively coupled plasma optical emission spectrometry (ICP-OES, Perkin Elmer instrument).

### CO Elimination Test

The detection of CO elimination was performed by IR with a JASCO FTIR-460 spectrometer (resolution 4 cm^−1^, integration 20 times) and measured at room temperature. A sample was placed in an IR gas cell with KBr windows, and no treatment was applied before the measurement of elimination activity. 0.5 g of CuMnO_x_@MS catalyst was used in the IR gas cell. CO gas (50 mL) was added to the IR gas cell. The IR spectrum was obtained every 10 min at room temperature. The schematic of the CO elimination efficiency evaluation setup composed of a JASCO FTIR-460 spectrometer is shown in Additional file 1: Figure S1.

## Results and Discussion

**Scheme 1:**
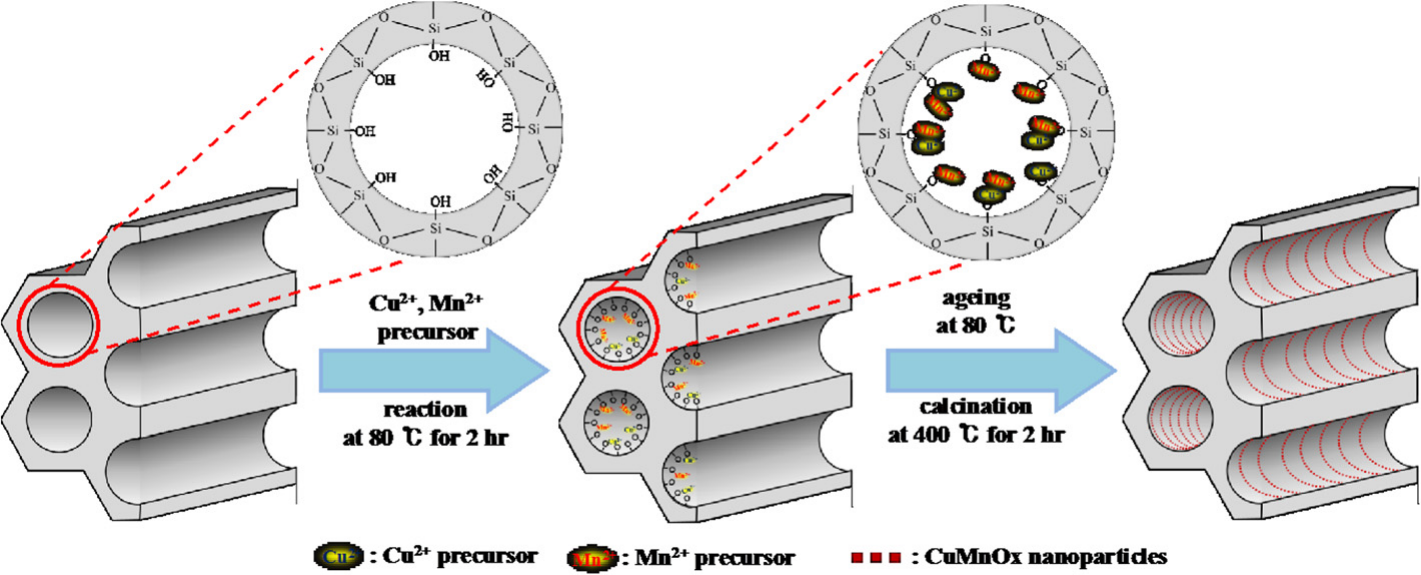
Preparation of binary CuMnO_x_@MS catalysts with various [Mn]/[Cu] concentrations

Scheme 1 shows the preparation of the CuMnO_x_@MS catalysts used for highly efficient CO removal, using the method of co-precipitation and calcination as a function of [Mn] concentration. The Cu-Mn metal precursors were impregnated into the pore channels and then calcined to metal oxide at 400 °C. This approach enables the facile development of binary CuMnO_x_ nanoparticles in the highly ordered mesopores, which results in effective CO removal

.

**Fig. 1:**
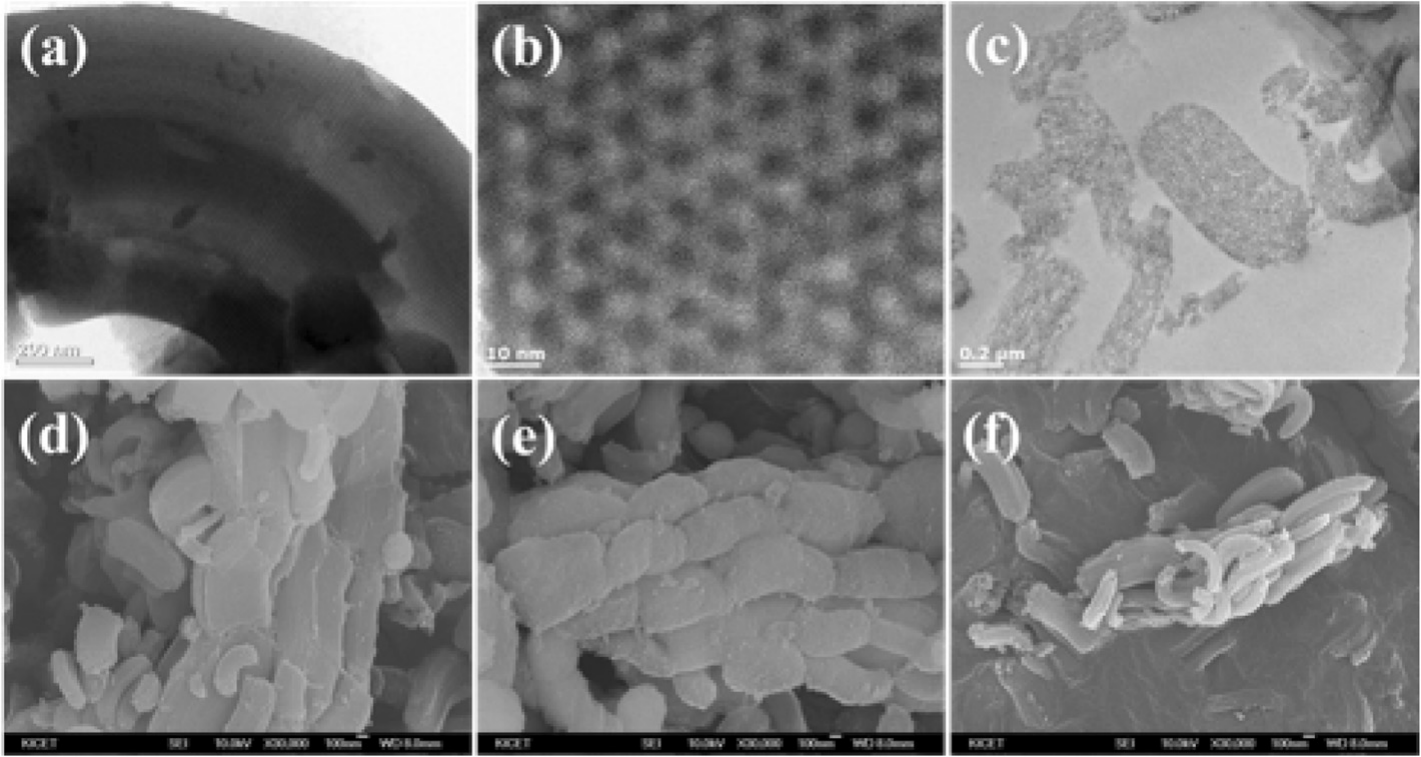
TEM images of **a** mesoporous silica and **b**, **c** CuMnO_x_@MS-4. FE-SEM images of **d** CuMnO_x_@MS-1, **e** CuMnO_x_@MS-2, and **f** CuMnO_x_@MS-4

TEM and FE-SEM images were obtained to investigate the morphology and diameter of the binary CuMnO_x_ particles. The TEM images (Fig. 1a) of mesoporous silica show the highly ordered arrangement of the pore channels and reveal the hexagonally ordered channels and frameworks of CuMnO_x_@MS-4 as well as the binary metal oxides located inside the pores (Fig. 1b, c). With the increase of [Mn] concentration, ordered 2D hexagonal mesostructures can also be observed. These results mean that binary CuMnO_x_ nanoparticles were located in the mesoporous silica channels, and the CuMnO_x_ nanoparticles were highly stable because of the protection of the mesoporous silica channels. FE-SEM images (Fig. 1d, e, f) clearly confirm that the cylinder-like morphology of the as-prepared mesoporous silica was maintained for all the catalysts, indicating no evident damage to the framework during the calcination process for metal oxide impregnation. For the CuMnO_x_@MS catalysts, nanoparticles cannot be seen outside the mesopore channels. In addition, the degree of dispersion of the binary CuMnO_x_ nanoparticles in the mesoporous silica was further elucidated by EDX mapping (Additional file 1: Figure S2). The images show the distribution of binary CuMnO_x_ nanoparticles at a resolution of ~1 μm, and the uniform X-ray intensities of Cu and Mn signals in the sample CuMnO_x_@MS can be clearly observed.

**Fig. 2:**
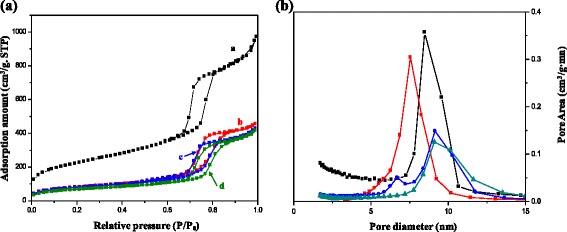
**a** Nitrogen adsorption-desorption isotherms and **b** pore-size distribution of the (a) highly ordered mesoporous silica, (b) CuMnO_x_@MS-1, (c) CuMnO_x_@MS-2, and (d) CuMnO_x_@MS-4

Nitrogen adsorption-desorption isotherms of mesoporous silica and CuMnO_x_@MS catalysts with different Mn contents are shown in Fig. 2a. All the isotherms were type IV curves with H1 type hysteresis loops [31, 32]. The preservation of the cylindrical mesostructures after the modification indicates the absence of structural damage of the mesoporous silica. With the increase in Mn contents, a significant decrease of the surface area was observed for the CuMnO_x_@MS catalysts, from 280 to 237 m^2^ · g^−1^, a decrease of pore volume from 0.71 to 0.64 cm^3^ · g^−1^, and pore diameter from 10.06 to 10.89 nm. Compared with the bare mesoporous silica (surface area 789 m^2^ · g^−1^, pore volume 0.64 cm^3^ · g^−1^, and pore diameter 7.82 nm), these results confirm the successful incorporation of metal oxide inside the mesoporous silica pore channels. With increasing Mn contents, the pore size distributions become wider, especially for CuMnO_x_@MS-4 with the highest Mn content (Fig. 2b). The nitrogen adsorption-desorption isotherm data are listed in Table 1. Furthermore, for a quantitative analysis of the binary CuMnO_x_ species formed in the mesopores at various Mn contents, ICP-OES measurements were carried out through titration. The back-titrated method employed was a modified version of a method reported in the literature [34]. The samples were dissolved in a HF/H_3_PO_4_ mixture by heating at 60 °C. Table 1 and Additional file 1: Figure S3 show that the ratio of weight percentage of [Mn]/[Cu] detected in the CuMnO_x_@MS catalysts was similar to the ratio values used in the synthesis solutions. The highest content of Mn was estimated to be 6.18 wt.% for CuMnO_x_@MS-4. Based on Table 1, this indicates that as the Mn contents increase, the three samples from CuMnO_x_@MS-1 to CuMnO_x_@MS-4 show decreasing pore volume and surface area. This should be because of the existence of binary CuMnO_x_ nanoparticles in the mesostructure channels.

**Table 1:** Physicochemical properties of the mesoporous silica and CuMnO_x_@MS samples synthesized with different Mn contents

Sample	Weight percentage^a^			Pore diameter^b^ (nm)	Pore volume^c^ (cm^3^ · g^−1^)	*S*_BET_^d^ (m^2^ · g^−1^)
	Mn	Cu	Mn/Cu			
MS	-	-	-	7.82	1.50	789
CuMnO_x_@MS-1	4.15	6.59	0.6	9.12	0.71	280
CuMnO_x_@MS-2	5.97	4.95	1.2	9.13	0.66	272
CuMnO_x_@MS-4	6.18	2.53	2.4	10.22	0.64	237

^a^Weight percentage determined by ICP-OES

^b^Adsorption average pore size calculated by BJH method

^c^Total pore volume was estimated at a relative pressure

^d^Specific surface area computed using BET equation in relative pressure of *p*/*p*_0_ = 0.05–0.3

**Fig. 3:**
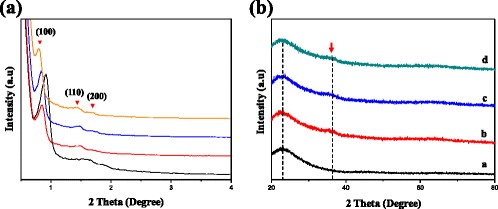
**a** SAXS and **b** XRD patterns of the (a) highly ordered mesoporous silica, (b) CuMnO_x_@MS-1, (c) CuMnO_x_@MS-2, and (d) CuMnO_x_@MS-4

The SAXS patterns for CuMnO_x_@MS catalysts with various [Mn]/[Cu] volume ratios are shown in Fig. 3a. As can be seen from this figure, all of the binary CuMnO_x_ nanoparticle-loaded mesoporous silica catalysts were highly ordered, with a 2D hexagonal framework, giving well-resolved peaks indexed as (100), (110), and (200) according to the p6mm symmetry group. Wide-angle X-ray diffraction (XRD) patterns (Fig. 3b) of CuMnO_x_@MS and MS were further carried out to study the crystal phases of the impregnated nanoparticles. They show quite similar diffraction peaks at around 23° and 38° for the CuMnO_x_@MS sample series with different Mn contents. The average crystalline sizes can be roughly calculated by the diffraction peaks. A broad peak centered at 23° of 2θ was observed for all samples, indicating that the mesostructures of MS and CuMnO_x_@MS are amorphous. At 38°, the peak was observed for the CuMnO_x_@MS sample series, indicating that the crystal phases of binary CuMnO_x_ were an amorphous type with a diffraction peak similar to hopcalite [35]. This may be because of the co-precipitation method or the high temperature calcination. TGA measurements (Additional file 1: Figure S4) showed that as the Mn contents increased, the three samples from CuMnO_x_@MS-1 to CuMnO_x_@MS-4 showed an increase in thermal stability and reduction of weight loss. This should be because of the synergistic effect of binary CuMnO_x_ nanoparticles in the mesostructure channels.

**Fig. 4:**
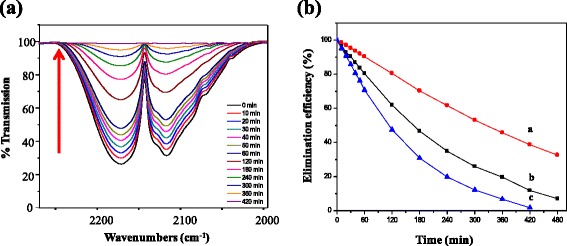
**a** IR spectra of the CuMnO_x_@MS-4 sample for elimination of gaseous CO as a function of reaction time with 0.5g catalyst, and **b** elimination efficiency with various CuMnO_x_@MS with different Mn contents at ambient temperature: (a) CuMnO_x_@MS-1, (b) CuMnO_x_ @MS-2, and (c) CuMnO_x_@MS-4

The catalytic property of the CuMnO_x_@MS was examined with an IR gas cell (with KBr windows, capacity of 50 cc, Additional file 1: Figure S1) containing CO gas at ambient temperature. Two major bands were seen at 2117 and 2171 cm^−1^ corresponding to *ν*_co_ stretching (Fig. 4a). Moreover, the intensity of gaseous CO peaks decreased gradually as a function of time. For the typical CuMnO_x_@MS-4 catalyst, over 98 % CO gas could be efficiently removed after 420 min at room temperature. The catalytic reaction rate as a function of time for the CuMnO_x_@MS catalysts was measured at room temperature (Fig. 4b). With increasing Mn contents, the catalytic activity slightly improved, exhibiting an increase in removal efficiency of 68, 82, and 98 % at 420 min for the CuMnO_x_@MS-1, CuMnO_x_@MS-2, and CuMnO_x_@MS-4, respectively.

## Conclusions

In this study, binary CuMnO_x_ nanoparticle-loaded MS catalyst was successfully synthesized by co-precipitation and demonstrated for CO elimination at room temperature. Based on detailed characterizations, including SAXS, BET, and HR-TEM techniques, the binary CuMnO_x_ nanoparticles were determined to be amorphous type with a diffraction peak similar to hopcalite. Moreover, the catalytic activity of the CuMnO_x_@MS catalysts was investigated for various [Mn]/[Cu] concentrations. With increasing [Mn] concentration, the catalytic activity was increased. Among these catalysts, CuMnO_x_@MS-4 showed the highest catalytic activity, of over 98 % CO elimination after 420 min at room temperature. The binary Cu-Mn metal oxide-loaded MS has good potential for practical applications to decrease CO in air pollution.

## Additional File

Additional file 1: Figure S1. Schematic of the CO elimination efficiency evaluation setup composed of a JASCO FT-IR-460 spectrometer. **Figure S2.** Face mapping images of CuMnOx@MS-.4. **Figure S3.** ICP analysis of Cu and Mn element contents in binary metal oxide. **Figure S4.** TG curves of binary CuMnOx nanoparticles impregnated MS catalysts synthesized various Mn contents: (a) CuMnOx@MS-1, (b) CuMnOx@MS-2, (c) CuMnOx@MS-4. (DOCX 1.83 mb)
