# Supplementary material for: Highly Efficient Elimination of Carbon Monoxide with Binary Copper-Manganese Oxide Contained Ordered Nanoporous Silicas
Source: Nanoscale Res Lett. 2016 Jan 7;11:6. doi: 10.1186/s11671-015-1197-4 (PMC4705076; doi:10.1186/s11671-015-1197-4)

**Supporting information**

**Highly Efficient Elimination of Carbon Monoxide with Binary Copper-Manganese Oxide contained Ordered Nanoporous Silicas**

Jiho Lee. Hwayoun Kim. Hye-Sun Lee. Seo-Jun Jang and Jeong-Ho Chang*

*Korea Institute of Ceramic Engineering and Technology, Jinju 660-031, Korea*

*Corresponding author. Tel: +82 55 792 2750; Fax: +82 55 792 2740

E-mail: [jhchang@kicet.re.kr](mailto:jhchang@kicet.re.kr)

**Fig. S1.** Schematic of the CO elimination efficiency evaluation setup composed of a JASCO FT-IR-460 spectrometer.


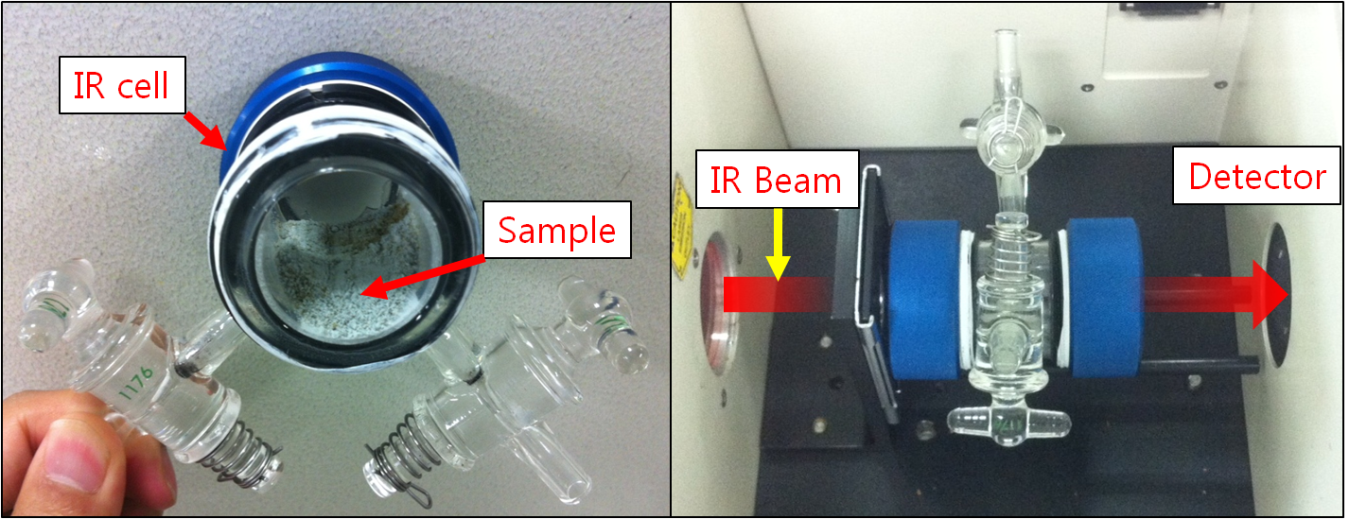


**Fig. S2.** Face mapping images of CuMnOx@MS-.4


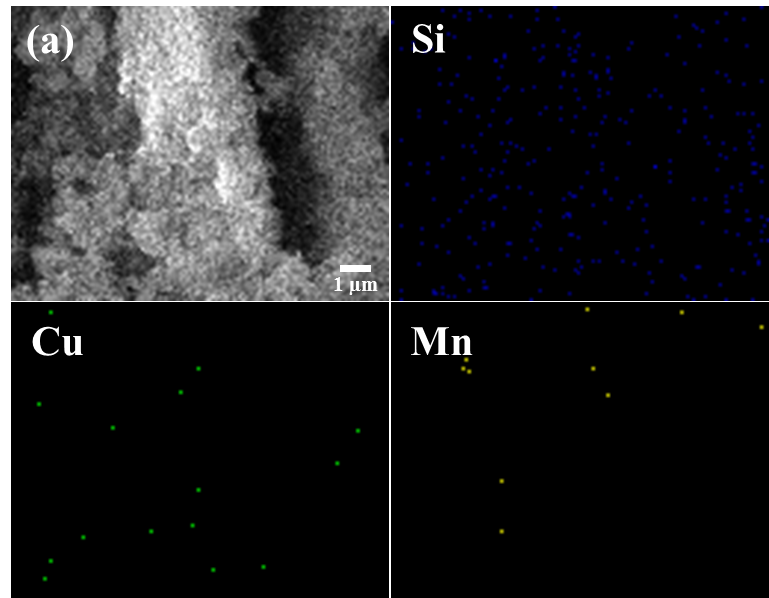


**Fig. S3.** ICP analysis of Cu and Mn element contents in binary metal oxide.


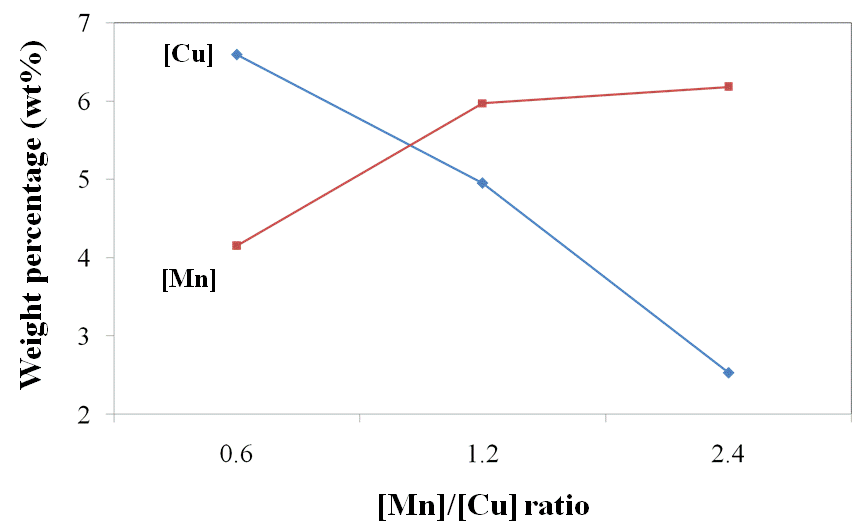


**Fig. S4.** TG curves of binary CuMnO_x_ nanoparticles impregnated MS catalysts synthesized

various Mn contents: (a) CuMnO_x_@MS-1, (b) CuMnO_x_@MS-2, (c) CuMnO_x_@MS-4.


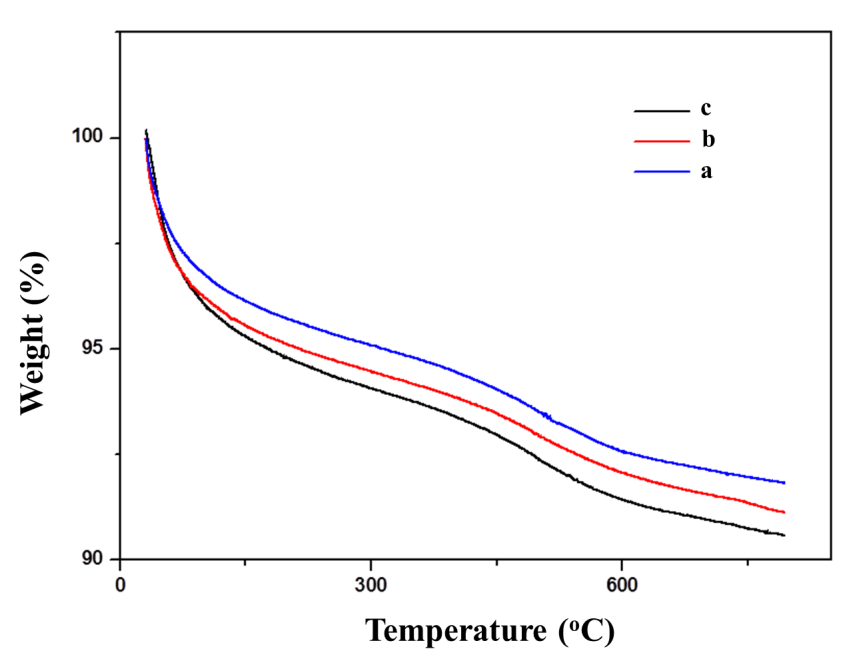

Supplement: Additional file 1: Figure S1. — Schematic of the CO elimination efficiency evaluation setup composed of a JASCO FT-IR-460 spectrometer. Figure S2. Face mapping images of CuMnOx@MS-.4. Figure S3. ICP analysis of Cu and Mn element contents in binary metal oxide. Figure S4. TG curves of binary CuMnOx nanoparticles impregnated MS catalysts synthesized various Mn contents: (a) CuMnOx@MS-1, (b) CuMnOx@MS-2, (c) CuMnOx@MS-4. (DOCX 1.83 mb) [file 11671_2015_1197_MOESM1_ESM.docx]
